# Familial Pancreatic Cancer Research: Bridging Gaps in Basic Research and Clinical Application

**DOI:** 10.3390/biom14111381

**Published:** 2024-10-30

**Authors:** Suyakarn Archasappawat, Fatimah Al-Musawi, Peiyi Liu, EunJung Lee, Chang-il Hwang

**Affiliations:** 1 Department of Microbiology and Molecular Genetics, College of Biological Sciences, University of California, Davis, Davis, CA 95616, USA; sarcha@ucdavis.edu (S.A.); falmusawi@ucdavis.edu (F.A.-M.); pyiliu@ucdavis.edu (P.L.);; 2 University of California Davis Comprehensive Cancer Center, University of California, Davis, Sacramento, CA 95817, USA

**Keywords:** familial pancreatic cancer, BRCA2, BRCA1, PARP inhibitor, pancreatic ductal adenocarcinoma, homologous recombination

## Abstract

Familial pancreatic cancer (FPC) represents a significant yet underexplored area in pancreatic cancer research. Basic research efforts are notably limited, and when present, they are predominantly centered on the *BRCA1* and *BRCA2* mutations due to the scarcity of other genetic variants associated with FPC, leading to a limited understanding of the broader genetic landscape of FPC. This review examines the current state of FPC research, focusing on the molecular mechanisms driving pancreatic ductal adenocarcinoma (PDAC) progression. It highlights the role of homologous recombination (HR) and its therapeutic exploitation via synthetic lethality with PARP inhibitors in BRCA1/2-deficient tumors. The review discusses various pre-clinical models of FPC, including conventional two-dimensional (2D) cell lines, patient-derived organoids (PDOs), patient-derived xenografts (PDXs), and genetically engineered mouse models (GEMMs), as well as new advancements in FPC research.

## 1. Introduction

Pancreatic cancer is one of the most aggressive and deadliest cancers. With a late diagnosis due to the lack of early-stage symptoms and a poor prognosis, it has a current 5-year survival rate of 13% and is estimated to become the second-leading cause of cancer-related mortality by 2030 [1,2]. The most common type of pancreatic cancer is termed pancreatic ductal adenocarcinoma (PDAC). It stems from the exocrine pancreas, which is primarily responsible for the secretion of digestive enzymes, ions, and water into the duodenum of the gastrointestinal tract. While surgical resection is the sole curative option, only around 15–20% of patients can undergo resection due to the early metastasis of PDAC, making the early detection of PDAC a necessary but challenging goal [3,4]. The National Comprehensive Cancer Network (NCCN) recommends either chemotherapy combinations, gemcitabine with nab-paclitaxel, or FOLFIRINOX (folinic acid, 5-FU, irinotecan, oxaliplatin) as first-line treatments [1]. However, clinical benefits from the standard chemotherapies for PDAC patients remain modest.

The progression of PDAC is prompted by somatic mutations of key driver genes. The prevalence of the somatic mutation in PDAC includes: activating mutations in *KRAS*, 90%; inactivating mutations in *TP53*, 50–74%; inactivating mutations in *CDKN2A*, 46–60%; and inactivating mutations in *SMAD4*, 31–38% [2]. Oncogenic mutations in *KRAS* in pancreatic ductal epithelial cells are known to initiate pancreatic intraepithelial neoplasia (PanIN), ultimately resulting in PDAC with subsequent mutations in other tumor suppressor genes. KRAS is a membrane-bound guanosine triphosphate (GTP) binding protein and mainly functions in cell growth and proliferation. CDKN2A plays essential roles in the expression and functionality of cell-cycle regulators, including p53 [3]. Furthermore, mutations in *TP53* and *SMAD4* are frequently detected in PanIN-3 and invasive tumors, which drives the expansion of pancreatic cancers [4]. In addition to these somatic mutations, a portion of patients with a family history of PDAC carry germline mutations, which increase their risk of developing PDAC, known as **familial pancreatic cancer (FPC)** [5].

## 2. Statistical Risk of Familial Pancreatic Cancer

Since its establishment in 1994, the National Familial Pancreas Tumor Registry (NFPTR) has been recruiting cases of FPC, which is defined as patients with two or more first-degree relatives diagnosed with pancreatic cancer. Patients without such a family history are classified as having sporadic pancreatic cancer (SPC) [6,7]. These definitions have become the consensus for FPC and SPC. FPC cases comprise approximately 10% of total pancreatic cancer cases [8]. Individuals with first-degree relatives diagnosed with pancreatic cancer are found to have a significantly higher risk of developing pancreatic cancer compared to the general population. The extent of pancreatic cancer risk is directly proportional to the number of affected first-degree relatives [7,8,9]. Having one diagnosed first-degree relative increases the risk by 4.5-fold, having two diagnosed first-degree relatives leads to a 6.4-fold increase, and having three or more diagnosed first-degree relatives increases the risk by 32-fold. However, the elevated risk is not observed in spouses and other genetically unrelated relatives, highlighting the significant role of genetic factors in the etiology of FPC [7].

A meta-analysis of seven case–control and two cohort studies, independently conducted in different geological locations and across a 40-year time span, verified that family history is a risk factor for pancreatic cancer, despite variations in location and methodology. This meta-analysis further supports previous findings, concluding that having more than one affected first or second-degree relative harbors a nearly twofold increased risk of pancreatic cancer [10]. In addition, when comparing successive generations within FPC families, each generation showed a lower age of death and a higher risk of pancreatic cancer death than its previous generation [11]. Individuals with family histories of pancreatic cancer also have higher risks of developing other types of cancers, including prostate cancer, liver carcinoma, lymphoma, and colon cancer [12]. These meta-analyses statistically demonstrate the familial aggregation of pancreatic cancer and suggest a strong correlation between genetic factors and the development of pancreatic cancers. Investigations on the genetic basis of FPC will provide critical support for evolving areas such as cancer screening, prevention, management, and genetic counseling for high-risk individuals. This has led to various studies aimed at pinpointing the genetic variants associated with FPC.

## 3. Genes Associated with Familial Pancreatic Cancer

Early studies have identified *BRCA2* as one of the most commonly mutated genes in FPC [13,14]. *BRCA1* and *BRCA2* encode for key proteins activated in the presence of DNA double-strand breaks (DSBs) and subsequently mediate the DNA repair pathway. Failures in repairing DNA DSBs lead to genome instability and the generation of disruptive and harmful mutations that cause severe diseases and cancers [15,16,17,18,19]. Consistent with this, individuals with mutations in *BRCA1* and *BRCA2* harbor a high risk of developing various types of cancer, including breast, ovary, prostate, esophagus, stomach, and uveal cancers [19,20]. By direct sequencing of constitutional DNA, Murphy et al. identified five (17.2%) deleterious *BRCA2* mutations in FPC patients [13]. In addition, in 26 European families that met the criteria of FPC, 12% of the families carried germline frameshift mutations in *BRCA2* (6672insT, 6819delTG, and 4075delGT) that resulted in a truncated and non-functional BRCA2 protein. Additional two families were identified with sequence variants of BRCA2, resulting in an overall 19% prevalence of *BRCA2* mutations in FPC [14]. Among Ashkenazi Jewish breast cancer patients, a higher *BRCA1* and *BRCA2* mutation rate was found to be associated with a family history of pancreatic cancer. In patients who had first-, second-, and third-degree relatives with pancreatic cancer, the mutation prevalence was 15.4%, 15.3%, and 8.6%, respectively [21].

The development of next-generation sequencing has enabled the identification of a wider range of pathogenic germline mutations that increase the carriers’ risk of being diagnosed with pancreatic cancer, including *ATM*, *BRCA1*, *BRCA2*, *MLH1*, *MSH2*, *MSH6*, *TP53*, *PALB2*, *PMS2*, *PRSS1*, *STK11*, and *CDKN2A* [12,22]. Roberts et al., using whole genome sequencing, confirmed the existing FPC susceptibility genes mentioned above and identified novel genes such as *BUB1B*, *CPA1*, *FANCC*, and *FANCG*. This study reported that 1077 genes were found to have two or more heterozygous premature truncating variants (loss-of-function mutations), demonstrating the high genetic heterogeneity within FPC. Notably, many top-hit candidate genes are involved in the DNA damage repair pathway and genome stability regulation, hereafter referred to as “FPC genes” [23]. Among these genes, *BRCA2* remained the most commonly mutated gene. Other studies employed nearly or more than 700 patient samples collected from multiple institute sites, without being limited to a single ethnicity. They tested and compared the prevalence of *BRCA1*, *BRCA2*, *PALB2*, and *CDKN2A* mutations, four of the top FPC-associated DNA repair gene mutations, in FPC and SPC patients (Table 1) [12,22,23]. These studies identified multiple novel *BRCA2* variants such as 6224insT, confirmed that FPC patients carry more mutations in the above four genes than SPC patients, and concluded that *BRCA2* and *CDKN2A* account for the majority of mutations within FPC. In a separate study comparing *ATM* mutants between the FPC and SPC groups, *ATM* mutants have a higher prevalence in FPC patients compared to the general population [24].

Taken together, these findings suggest that inheritable mutations in multiple genes involved in DNA damage repair and genome stability maintenance lead to a higher risk of developing pancreatic cancer, with *BRCA2* being the most commonly mutated gene compared to others in FPC.

## 4. Double-Strand Breaks (DSB) and Homologous Recombination (HR) Pathway

Since defects in FPC genes predispose individuals to the development of pancreatic cancer by disrupting the DNA damage response (DDR), a better understanding of how these contribute to the pathogenesis could help in exploiting parts of the pathways therapeutically. DNA can be damaged through exogenous and endogenous damages, leading to DSBs, which pose a serious threat to cell viability and genome stability. DSBs can be generated naturally when replication forks encounter blocking lesions such as those produced as a byproduct of cellular respiration, mainly reactive oxygen species (ROS) leading to fork collapse [26]. DSBs are also produced when cells are exposed to DNA damaging agents such as ionizing radiation, chemical agents, UV light, transposons, and replication of a region that has a nick in the backbone. DSBs can also occur during programmed genome rearrangements induced by nucleases, and during physical stress when chromosomes are pulled to opposite poles during mitosis [27]. The failure to properly repair DSBs can result in cell death or large-scale chromosome changes, including deletions, translocations, and chromosome fusions that enhance genome instability and are hallmarks of cancer [28].

Two major pathways, non-homologous end joining (NHEJ) and homologous recombination (HR), are used to repair DNA DSBs. NHEJ is an error-prone pathway in which the ends of the DSB are ligated back together nonspecifically, potentially causing insertions and deletions. On the contrary, HR is a high-fidelity pathway in which cells repair DSBs using a DNA template typically from the sister chromatid. When using the sister chromatid strand as a template, HR can result in a loss of heterozygosity with information transferred non-reciprocally from the unbroken donor locus to the broken recipient locus in a process called gene conversion [29]. HR occurs during the S and G2 phases of the mammalian cell cycle when the homologous chromosome or the sister chromatid is available due to CDK-dependent phosphorylation of CtIP, a factor known to stimulate end resection [30]. CtIP also prevents the diploid cell from using the sister chromatid as a template for repair, which can cause a loss of heterozygosity [31].

**Figure 1:**
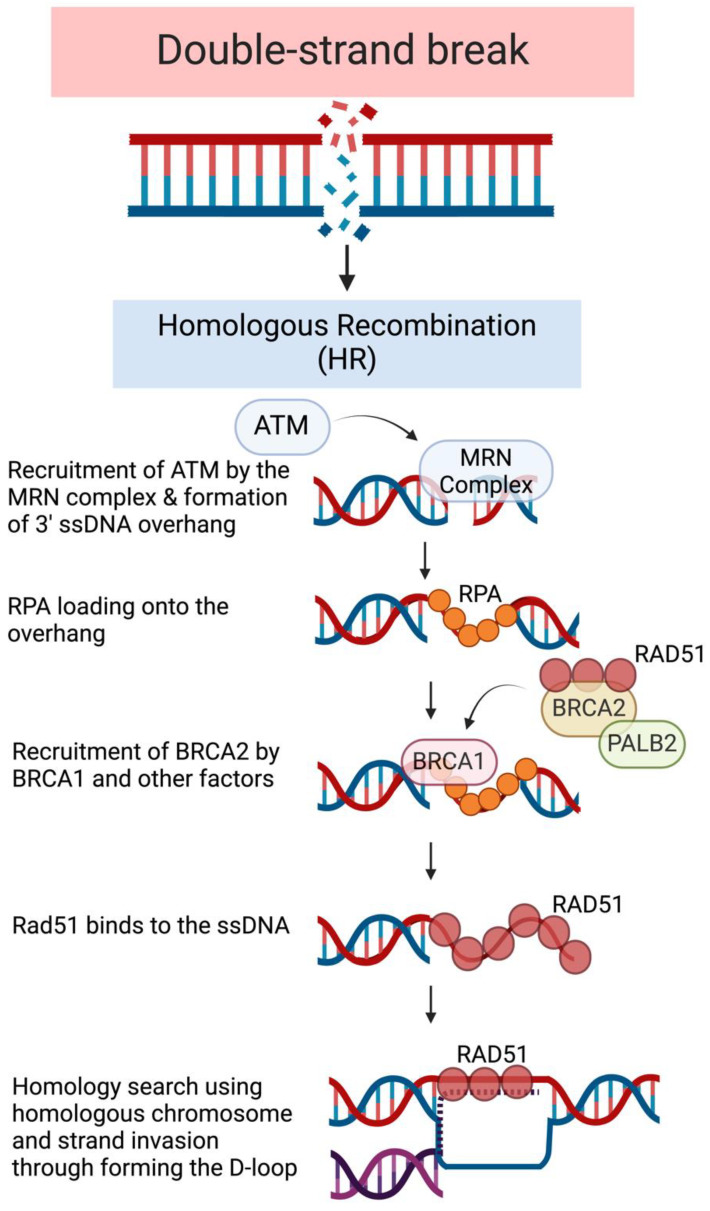
Steps of homologous recombination. Homologous recombination (HR) repairs double-strand breaks (DSB) in three key steps. First, during pre-synapsis, the MRN complex processes the DNA ends to generate 3′ single-stranded DNA (ssDNA) overhang and recruits ATM kinase. During synapsis, RPA then binds the overhang, and BRCA1 is recruited to the site. BRCA1 then facilitates the recruitment of BRCA2, and PALB2, along with RAD51, mediates the invasion of the homologous DNA duplex to form a displacement loop (D-loop). Finally, DNA polymerases extend the invading strand using the homologous template, completing the repair process.

HR deficiency is particularly relevant to FPC due to BRCA1/2’s involvement in HR, as they are the most commonly mutated genes. HR is carried out by three main steps starting when HR is triggered from a DDR signal cascade that is aided by DDR proteins (Figure 1). The first step of the HR pathway, termed “pre-synapsis”, involves DNA end-resection to generate a 3′ ssDNA overhang. The DSB signaling is initiated via the binding of the MRN complex (MRE11, RAD50, and NBS1) to the broken DNA ends. The MRN complex facilitates endonucleolytic cleavages near the DSB towards the DNA end in 3′-5′ direction from the nick site to generate a 3′ ssDNA overhang and recruit endonuclease ExoI or helicase BLM to perform bulk 5′-3′ DNA resection. The MRN complex also plays a critical role in recruiting and activating ATM at DSB sites to orchestrate the repair process [32]. The second step, termed “synapsis”, consists of a homologous search and DNA strand invasion. Replication protein A (RPA) is then loaded onto the ssDNA by the MRN complex, which preserves the integrity of the resultant ssDNA [33]. In cells, BRCA2 can be recruited to DSBs by BRCA1 and PALB2 [32]. RPA, with the help of BRCA2, can recruit RAD51 recombinase, a DNA-dependent ATPase that serves as the main catalyst involved in DNA strand invasion repair. RAD51, recruited by BRCA2, then competes with RPA to gain access to the ssDNA and initiate HR-directed repair [32,34]. After recruitment, RAD51 forms a nucleoprotein filament with ssDNA, which promotes strand invasion and displacement loop (D-loop) formation [31,34]. The D-loop allows the 3′ end of the invading strand to prime DNA synthesis of the template duplex DNA [35]. The last step is “strand extension”. In this stage, DNA polymerase elongates the invading strand using the homologous template strand. After strand synthesis, the intermediate structures are resolved, and DNA ligases seal the nicks in the newly synthesized DNA to complete the repair, finally resulting in repaired DNA [34].

How genetic predispositions in FPC genes promote PDAC progression and how we can exploit these genetic defects therapeutically are active research areas. One promising approach is the concept of synthetic lethality between HR defects and Poly(ADP-ribose)-polymerase (PARP) inhibition. In BRCA1/2-deficient pancreatic cancer, defects in HR DNA damage repair create vulnerabilities that can be targeted by chemotherapies or targeted therapies. PARP is an enzyme involved in DNA base excision repair. The concurrent loss of the BRCA1/2-mediated HR pathway and PARP pathway results in an excessive accumulation of DNA damage, thereby leading to synthetic lethality. Indeed, BRCA1/2-deficient tumor cells are more sensitive to PARP inhibition, thus making PARP inhibition an effective treatment strategy for BRCA1/2-deficient pancreatic cancer [36]. BRCA1/2-deficient tumor cells are also more sensitive to platinum-based chemotherapies and anthracyclines, which are selectively lethal in HR-deficient cells [37]. According to the NCCN guidelines, genetic testing is currently recommended for all PDAC patients, partly because HR defects can benefit from PARP inhibition [1]. However, other HR-related gene defects require more epidemiological and molecular evidence to provide more informed and comprehensive genetic counseling and guidelines for both FPC patients and those at elevated risk of pancreatic cancer.

## 5. FPC and PARP Inhibitors

Previous research has illuminated the pivotal role of *BRCA* genes in maintaining genomic stability, with mutations leading to defective DNA repair mechanisms. The defect in the DNA repair process conferred increased sensitivity to DNA-damaging agents like platinum-based chemotherapy and, more importantly, PARP inhibitors. PARP inhibitors emerged as a novel class of drugs exploiting the concept of synthetic lethality, wherein *BRCA*-mutated cancer cells deficient in DNA repair are particularly susceptible to PARP inhibition [38]. This strategy has been effective in treating *BRCA*-mutant cancers, including breast and ovarian cancers, as well as pancreatic cancer. In particular, the Pancreas Cancer Olaparib Ongoing (POLO) clinical trial demonstrated that olaparib significantly extended progression-free survival (PFS) compared to placebo in patients with *BRCA*-mutant pancreatic cancer following platinum-based chemotherapy [39]. Other PARP inhibitors like rucaparib [40], talazoparib [41,42,43], and the novel AZD 5305 have shown promise in clinical trials, indicating the potential of PARP inhibition in targeted cancer therapy, particularly for *BRCA*-mutated tumors, by exploiting their reliance on specific DNA repair pathways [44].

Subsequent studies expanded the utility of PARP inhibitors, exploring various combinations and settings, with other PARP inhibitors like rucaparib and veliparib also being investigated for their efficacy in *BRCA*-mutated cancers. In 2020, a study by O’Reilly and colleagues explored the combination of gemcitabine, cisplatin, and veliparib for *BRCA*-mutated PDAC, finding only a marginal improvement in response rates without statistical significance, with veliparib reducing PFS and increasing adverse events [45]. Concurrently, PARP inhibitors have been recognized for enhancing PD-L1 expression and stimulating neoantigen formation, prompting investigations such as the SWOG S2001 trial, which examines the combination of olaparib with pembrolizumab [46]. Early trials, such as those combining novel BET and WEE1 kinase inhibitors with PARP inhibitors, showed potential synergies and explored safety and efficacy in human studies [47], highlighting the ongoing search for effective combinations and novel therapeutic strategies. Currently, the NCCN guidelines’ recommendation of olaparib as maintenance therapy for patients with germline *BRCA1/2* mutations following platinum-based chemotherapy has established it as a standard of care, leveraging precision medicine to improve outcomes by targeting genetic vulnerabilities in pancreatic cancer [48].

PARP inhibitors are a crucial treatment strategy for patients with *BRCA*- or *PALB2*-mutated pancreatic cancer, offering a valuable maintenance therapy option. These inhibitors exploit the cancer cells’ compromised DNA repair mechanism, leading to selective cancer cell death. However, the effectiveness of PARP inhibitors can be undermined by the development of resistance, a significant hurdle in the long-term management of the disease. One notable mechanism of PARP inhibitor resistance is the emergence of BRCA reversion mutations [49]. These mutations restore the function of the *BRCA* gene, enabling cancer cells to regain their DNA repair capabilities and resist PARP inhibitor treatment. The presence of these reversion mutations is associated with a faster progression of the disease post-PARP inhibitor therapy and a decreased overall survival rate [50,51,52]. Brown et al. examined advanced, platinum-sensitive pancreatic cancer patients treated with rucaparib. They found that acquired reversion mutations in *BRCA* or *PALB2* were relatively rare, but profoundly impacted treatment outcomes [53]. While *BRCA* reversion mutations upon PARP inhibitors or platinum therapies appear to be common in breast and ovarian cancer patients, these are not commonly observed in pancreatic cancer [54,55]. In the patient cohort under study by Brown et al., only a minority exhibited these reversion mutations, indicating that while they are influential, they do not account for all cases of PARP inhibitor resistance. This rarity underscores the complexity of resistance mechanisms and the need for ongoing research to understand and overcome these challenges.

In summary, while PARP inhibitors offer a promising therapeutic avenue for *BRCA*-mutated or HR-deficient pancreatic cancer, resistance remains a critical issue, with *BRCA* reversion mutations playing a significant but uncommon role. Identifying these mutations can provide valuable insights into the patient’s prognosis and guide subsequent therapeutic strategies, emphasizing the need for personalized approaches in treating pancreatic cancer.

## 6. Pre-Clinical Models of FPC

The limited efficacy of PARP inhibitors and the emergence of resistance mechanisms underscore the importance of understanding precise molecular mechanisms driving FPC development and progression. Current research understandably focuses on *BRCA1/2* mutations, as these alterations are the most prevalent among FPC patients. However, mutations in FPC susceptibility genes, although relatively rare, also warrant attention. Therefore, this section reviews available pre-clinical models of FPC, examining their strengths and limitations in advancing our understanding of this complex disease (Table 2). A comprehensive review of pre-clinical models of PDAC is beyond the scope of this review and has been discussed elsewhere [56]. Here, we will review pre-clinical models specifically relevant to FPC.

In vitro models play an important role in this endeavor, with conventional cell lines offering a controlled experimental setting for dissecting the intricate genetic and cellular aberrations characteristic of the disease. Among these, the CAPAN-1 cell line commonly serves as a representative model for studying BRCA2-deficient PDAC. The cell line possesses a naturally occurring frameshift mutation in *BRCA2* (c.6147delT), which generates a premature stop codon. This genetic alteration causes a pathogenic frameshift mutation (p.S1982fs*22), leading to truncating critical C-terminal amino acids of the BRCA2 protein and compromising HR repair [57]. Despite their utility, conventional two-dimensional cell line models have significant limitations. These models often lack isogenic controls, complicating comparisons of drug responses across different cell lines due to additional genetic mutations and varying cellular contexts. Moreover, these models fail to accurately recapitulate the three-dimensional cellular architecture found in tumors, potentially skewing signaling and cellular behaviors [58]. In response to these challenges, patient-derived organoids (PDO) have emerged as a novel pre-clinical model. PDOs better mimic the pathophysiology of the originating tumors. For instance, a study by Tiriac et al. utilized PDOs as a primary experimental platform to explore the interplay between genomic alterations and drug sensitivity, although the authors did not observe a significant association between sensitivity to PARP inhibitors and mutations in HR-related genes [59]. Importantly, the PDO library used in the study did not exhibit deleterious mutations in these genes, nor did it show bi-allelic mutations in *BRCA1/2*. Therefore, this underscores the necessity for a larger and more diverse PDO library, specifically including samples from FPC cases, to comprehensively address these research questions.

In addition to PDOs, patient-derived xenograft (PDX) and patient-derived organoid xenograft (PDOX) models also preserve the genetic heterogeneity and histological features of the patient’s tumor, making them a powerful tool for investigating therapeutic responses in vivo. Studies have shown that these models, harboring mutations in FPC genes, can precisely predict patient responses to various cancer treatments, including those targeting DNA damage repair pathways [60,61]. However, the predicted treatment response does not seem to be based solely on FPC gene mutations. Golan et al. observed diverse responses among *BRCA1/2*-mutant PDXs to DNA damaging agents, which reflect the wide spectrum of clinical responses seen in patients [62]. This variability likely stems from factors such as the PDX collection sites, *BRCA* status in PDX (heterozygous vs. loss of heterozygosity), and prior exposure to platinum-based or PARP inhibitor treatments. More importantly, the absence of a functional immune system in these models could further complicate predictions since PDX and PDOX lack immune cells, a critical component of the tumor microenvironment (TME), which might affect drug responses. Given the growing significance of immunotherapy in cancer treatment, it is crucial to evaluate drug responses and immune-targeting strategies in models with intact immune systems, such as genetically engineered mouse models (GEMMs). These models can provide a more comprehensive understanding of tumor-immune interactions and therapeutic efficacy, bridging the gap left by PDX and PDOX models.

GEMMs, with their intact immune system, provide a sophisticated means to study the genetic complexity and disease progression of FPC from early precursor lesions to metastasis. These models are particularly valuable as they incorporate the critical mutations driving PDAC, including the gain-of-function mutation in *Kras* together with the loss-of-function mutation in *Trp53* [63]. In PDAC GEMMs, these mutations are introduced as germline alterations and only activated within pancreatic epithelial cell lineage using a pancreas-specific Cre recombinase (*Pdx1-Cre*). Prominent examples of such models include the KC (*Kras*^+*/LSL-G12D*^; *Pdx1-Cre*) and KPC (*Kras*^+*/LSL-G12D*^; *Trp53*^+*/LSL-R172H*^; *Pdx1-Cre* or *Kras*^+*/LSL-G12D*^; *Trp53*^+*/LSL-R270H*^; *Pdx1-Cre*) mouse lines, which resemble the development of PanIN and its progression to PDAC [64]. GEMMs’ robust representation of disease progression, coupled with their ability to model DDR deficiencies seen in FPC gene contexts, makes them indispensable for researching FPC biology. These models typically employ conditional knock-out alleles of FPC susceptibility genes, such as *Brca1*, *Brca2*, *Atm*, and *Palb2*, in the KC or KPC background. Studies have shown that homozygous knock-out of these FPC genes significantly accelerates PDAC progression [65,66,67,68,69,70]. These studies confirm that DNA repair deficiency contributes to PDAC progression and provides a mechanistic insight into how these mutations predispose PDAC development in FPC patients. The heterozygous knock-out model of these genes also displayed an intermediate level of PDAC progression, likely due to the dose-dependent impact of these genetic mutations on cancer development [66,67,68,69,70]. Interestingly, the tumors from FPC heterozygous knock-out models retain the wild-type allele of the respective FPC genes, indicating that the loss-of-heterozygosity (LOH) of FPC genes is not an obligatory step in GEMMs [70]. However, this appears to be inconsistent with clinical observations, where a significant proportion of FPC tumors exhibit LOH of these genes [71]. The bi-allelic loss of FPC genes, as seen in clinical cases, appears to be critical for responses to PARP inhibitors or platinum-based drugs due to the compromised DNA repair capabilities of the tumor cells [72,73,74]. While it has clearly been shown in FPC GEMMs that FPC gene mutations promote PDAC progression in the *Kras* or *Kras*/*Trp53* mutant background, it remains to be addressed whether these FPC gene mutations directly co-operate with key driver mutations in PDAC patients, or whether these mutations facilitate the arrival of key driver somatic mutations due to impaired DNA repair. The latter can be supported by the observation that FPC patients exhibit somatic mutation profiles very similar to those seen in SPC [75,76,77,78]. It is possible that both ideas may contribute to PDAC progression to varying extents. Therefore, the detailed roles of genetic variants associated with FPC need to be further dissected using GEMMs, particularly in PDAC progression and response to therapy.

In sum, while no single model perfectly replicates the complexity of FPC, each plays a pivotal role based on the research objectives and questions. For drug screening and testing, cell lines and PDO models are indispensable, forming the backbone of preclinical studies that facilitate the translation of scientific discoveries like PARP inhibitors, which have successfully transitioned into clinical use. Moreover, GEMMs, in particular, provide unparalleled insights into FPC progression by closely mimicking the genetic and TME dynamics, potentially uncovering new therapeutic targets and vulnerabilities. These models remain essential for advancing our understanding of FPC and driving the development of innovative and more effective treatment strategies.

**Table 2:** Comparison of pre-clinical models used in familial pancreatic cancer research, highlighting their advantages and limitations. Models include conventional cell lines, patient-derived organoids (PDO), patient-derived orthotopic xenografts (PDOX), patient-derived xenografts (PDX), and genetically engineered mouse models (GEMM). These models vary in their abilities to recapitulate tumor complexity, genetic heterogeneity, and the tumor microenvironment (TME), with different suitability for drug screening, genetic manipulation, and immune studies.

Pre-Clinical Models	Advantages	Disadvantages	References
Conventional cell lines	Well-characterized Cost-effective Easy to maintain and genetically manipulate Homogenous population Rapid and scalable for preliminary studies Ideal for high-throughput drug screening and testing drug resistance	Lack of isogenic controls Simplistic representation without 3D structure, cell polarity, or complex interactions (e.g., cell–cell and cell–stroma interactions) Genomic drift due to clonal selection and adaptation to 2D conditions during repeated passaging introduces variability	[57]
Organoids and patient-derived organoids (PDO)	Higher success rate in establishing PDOs compared to 2D cell lines Relatively easier to establish/maintain PDOs than PDX models Retain 3D architecture Can reflect patient-specific tumor characteristics	Limited availability of FPC-specific PDOs for study Small PDO libraries make it challenging to capture the full mutation spectrum	[57,59]
Patient-derived organoid xenografts (PDOX)	Provide a versatile platform between in vitro and in vivo Allow genetic manipulation for personalized or targeted research	Share limitations with both PDO and PDX models, including variability from patient samples and limited immune system representation in immunocompromised models	[61]
Patient-derived xenografts (PDX)	Preserve patient-specific genetic heterogeneity and histological features Reflect clinical drug response more accurately than in vitro models	Require immunodeficientimmunocompromised mice, limiting immune interaction studies High inter-patient variability may complicate data interpretation (e.g., site of xenografts and patient’s treatment history)	[60,62]
Genetically engineered mouse models (GEMM)	Feasible for precise genetic engineering Ideal for studying cancer progression Feasible for TME studies due to intact immune system	Time- and resource-intensive Require longer study periods to observe relevant cancer progression Limited availability of FPC gene models	[65,66,67,68,69,70]

## 7. Current Status of Basic Research on FPC

FPC research has primarily focused on clinical and association-based studies, with little effort for mechanistic, basic research on the disease. Not surprisingly, most advancements have been made on *BRCA1/2* in the context of FPC. Although there has been progress in identifying genetic drivers of FPC, translating this knowledge into effective clinical practice remains a critical challenge. Such a challenge is compounded by the rarity of certain genetic variants and the pleiotropic roles of FPC genes, such as the HR-independent functions of BRCA1/2, which we are still in the process of understanding. Additionally, the significant influence of the TME on disease progression and treatment response adds to the complexity. Here, we discuss recent advancements in FPC research, highlighting key developments in understanding the genetic landscape, the creation of novel therapeutic strategies, and the novel roles of *BRCA* genes beyond HR, particularly their impact on the TME and potential for therapeutic exploitation.

Recent studies have focused on exploiting the unique vulnerabilities of the stroma in PDAC, aiming to develop novel therapies that target the dense and complex TME. PDAC is well-known for its highly dense desmoplastic stroma, which acts as a physical barrier, hindering immune cell penetration and contributing to its classification as a “cold tumor” [79]. Shaashua et al. explored the unique stromal landscape in *BRCA*-mutated PDAC and found an elevated activation of HSF1 in the stroma of *BRCA*-mutated PDAC. This activation drives the transcriptional regulation of clusterin (CLU), resulting in the up-regulation of immune-regulatory CLU-positive cancer-associated fibroblasts. This study suggests that this distinct stromal composition, characterized by HSF1-mediated *CLU* expression, could be a potential therapeutic target in BRCA-mutated PDAC [80]. Exciting advancements in cancer immunotherapy are focused on converting “cold” tumors into “hot” tumors, allowing for immune cell infiltration and improved treatment response. Oh et al. identified POLQ, a key mediator in the microhomology-mediated end joining (MMEJ) pathway, as a crucial pathway for DSB repair in BRCA2-deficient PDAC. POLQ inhibition represents a synthetic lethal approach to blocking tumor growth while concurrently activating the cGAS-STING signaling pathway, enhancing tumor immune infiltration and offering a novel therapeutic strategy [81]. Thus, these findings raise the possibility of targeting the unique aspects of the TME in *BRCA*-mutated PDAC to improve therapeutic outcomes.

As part of efforts to identify novel vulnerabilities of *BRCA2*-mutant PDAC, our group performed a high-throughput drug screening and discovered that the Bromodomain and Extra-Terminal domain protein (BET) inhibitors were particularly effective [82]. This heightened sensitivity in BRCA-deficient cells is linked to enhanced autophagic flux, a catabolic process of self-degradation and recycling to maintain cellular homeostasis. The increased autophagic flux is further elevated by BET inhibition, resulting in autophagy-dependent cell death. BET inhibitors have also been shown to be preferentially cytotoxic in the mutant *BRCA2* context in both breast cancer and pan-cancer settings in a publicly available database for Genomics of Drug Sensitivity in Cancer [83,84]. Consistent with these findings, Arun et al. also observed that knocking down *BRCA1/2* in triple-negative breast cancer cells induced autophagy, as evidenced by increased LC3-II expression—a key autophagy marker [85]. Together, these results suggest that BRCA2 may play a role as a negative regulator of autophagy. Counterintuitively, autophagy inhibition has been used to sensitize cells to drug responses. For instance, the sensitivity to cisplatin in BRCA2-depleted ovarian cancer cells was further sensitized by blocking autophagy with chloroquine [86]. These findings suggest a nuanced therapeutic potential in combining BET inhibition with strategies to modulate autophagy in BRCA2-deficient cancers.

Recent advances in cancer research have shed light on the HR-independent functions of BRCA2, such as epigenetic regulation and transcription control, albeit not in the PDAC context. Gruber et al. revealed that BRCA2 loss triggers a cascade of events leading to NF-kB signaling activation and increased acetylation of histone 4 (H4), affecting gene expression and cellular phenotype [87]. In addition, BRCA2 has been shown to resolve R-loops to prevent genome instability. R-loop is a specific DNA-RNA hybrid with displaced single-stranded DNA caused by nascent RNA re-annealing to its DNA template during transcription [88]. Studies have shown that BRCA2 associates with the TREX2-mRNA export factor PCIID2, preventing R-loop accumulation, and facilitates the transition from promoter-proximal pausing to productive elongation of transcription by recruiting PAF1 to PolII [89]. Additionally, BRCA2 recruits DDX5 helicase to the DNA damage site, further aiding in R-loop resolution [90]. These functions of BRCA2 appear to be critical for maintaining genomic stability, preventing transcription–replication conflicts, and modulating cellular responses. Elevated R-loop formation and higher autophagic flux in BRCA2-deficient cells can activate the cGAS-STING pathway, potentially leading to chronic cellular stress and influencing tumor development and immune surveillance.

Whether or not these findings in non-PDAC contexts can be applied to PDAC remains to be addressed, but they offer promising directions for future research in FPC. Understanding these multifaceted roles of BRCA2 offers new avenues for the personalized medicine approaches for FPC. As BRCA2’s non-HR functions can contribute to FPC pathogenesis, it is highly likely that other FPC genes also have multiple functions in various cellular processes, adding to the complexity of FPC biology. Further research is needed to dissect these detailed roles and exploit these genetic vulnerabilities.

## 8. Conclusions

**Figure 2:**
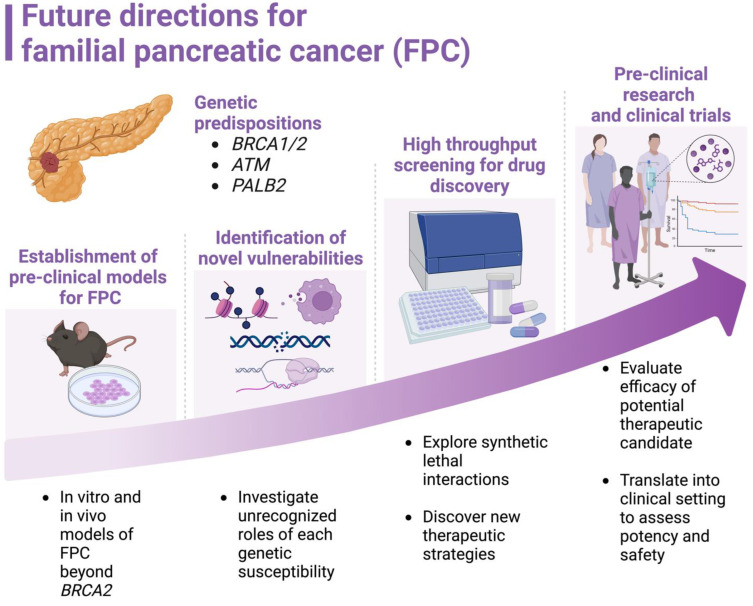
Future directions for familial pancreatic cancer (FPC). This figure presents a roadmap for future advancements in FPC, highlighting key areas of focus to improve patient outcomes. It emphasizes a multi-pronged approach encompassing the establishment of robust FPC models, identification of new vulnerabilities, personalized medicine strategies, and the application of translational research.

FPC represents a significant yet underexplored area within pancreatic cancer research. Overall, there is a lack of basic research on FPC. Among these, most studies have predominantly centered on the *BRCA1* and *BRCA2* mutations due to the scarcity of other FPC gene defects. This narrow focus has led to a limited understanding of the broader genetic landscape of FPC, thereby hindering the development of comprehensive therapeutic strategies. Advancing our understanding of FPC gene defects holds immense therapeutic potential. Exploiting the unique genetic makeup of FPC can lead to the development of targeted treatments, such as PARP inhibitors for *BRCA*-mutated tumors. However, the emergence of resistance mechanisms, including *BRCA* reversion mutations, underscores the necessity for ongoing research to identify and overcome these barriers. Furthermore, exploring the roles of other DDR-related genes and their pathways in FPC could unveil novel therapeutic targets. Investigating the HR-independent functions of BRCA2, such as R-loop resolution, transcription regulation, and replication fork protection, may provide additional avenues for intervention. The concept of “BRCAness,” where non-BRCA-deficient tumors exhibit similar vulnerabilities, also opens new possibilities for therapeutic exploitation through epigenetic modulation. Therefore, addressing the gaps in FPC research requires a multi-faceted approach. Expanding the focus beyond *BRCA1/2* mutations, developing diverse preclinical models, and leveraging the genetic intricacies of FPC gene defects will be crucial. A better understanding of FPC will pave the way for personalized medicine strategies that improve outcomes for FPC patients (Figure 2).

## Figures and Tables

**Table 1:** Prevalence of deleterious mutations in the genes *BRCA1*, *BRCA2*, *PALB2*, and *CDKN2A* in both FPC and SPC patients [22], as well as malignancies/disorders associated with the patients.

Gene	Prevalence of Deleterious Mutations		Other Associated Malignancies and Disorders
	FPC Patients	SPC Patients	
*BRCA1*	1.2%	0.0%	Breast, ovarian, prostate, esophageal, liver, stomach, uterine cancers [25]
*BRCA2*	3.7%	3.0%	Breast, ovarian, prostate, esophageal, pharyngeal, stomach, bone, gall bladder cancers, and melanoma [25]
*PALB2*	0.6%	0.5%	Fanconi anemia, breast, prostate, stomach, and esophageal cancers [6]
*CDKN2A*	2.5%	0.0%	Melanoma [6]
Total	8.0%	3.5%	
